# Applying federated learning to combat food fraud in food supply chains

**DOI:** 10.1038/s41538-023-00220-3

**Published:** 2023-09-01

**Authors:** Anand Gavai, Yamine Bouzembrak, Wenjuan Mu, Frank Martin, Rajaram Kaliyaperumal, Johan van Soest, Ananya Choudhury, Jaap Heringa, Andre Dekker, Hans J. P. Marvin

**Affiliations:** 1 Industrial Engineering & Business Information Systems, University of Twente, Enschede, The Netherlands; 2 Wageningen Food Safety Research, Akkermaalsbos 2, 6708 WB Wageningen, The Netherlands; 3 Information Technology Group, Wageningen University and Research, Wageningen, The Netherlands; 4 Netherlands Comprehensive Cancer Organization (IKNL), Eindhoven, The Netherlands; 5 Department of Human Genetics, Leiden University Medical Center, Leiden, The Netherlands; 6 Brightlands Institute for Smart Society, Faculty of Science and Engineering, Maastricht University, Heerlen, The Netherlands; 7 Department of Radiation Oncology (Maastro), GROW School for Oncology and Reproduction, Maastricht University Medical Centre, Maastricht, The Netherlands; 8 Centre for Integrative Bioinformatics (IBIVU), VU University Amsterdam, Amsterdam, The Netherlands; 9 Department of Research, Hayan Group, Rhenen, The Netherlands

**Keywords:** Risk factors, Chemical safety

## Abstract

Ensuring safe and healthy food is a big challenge due to the complexity of food supply chains and their vulnerability to many internal and external factors, including food fraud. Recent research has shown that Artificial Intelligence (AI) based algorithms, in particularly data driven Bayesian Network (BN) models, are very suitable as a tool to predict future food fraud and hence allowing food producers to take proper actions to avoid that such problems occur. Such models become even more powerful when data can be used from all actors in the supply chain, but data sharing is hampered by different interests, data security and data privacy. Federated learning (FL) may circumvent these issues as demonstrated in various areas of the life sciences. In this research, we demonstrate the potential of the FL technology for food fraud using a data driven BN, integrating data from different data owners without the data leaving the database of the data owners. To this end, a framework was constructed consisting of three geographically different data stations hosting different datasets on food fraud. Using this framework, a BN algorithm was implemented that was trained on the data of different data stations while the data remained at its physical location abiding by privacy principles. We demonstrated the applicability of the federated BN in food fraud and anticipate that such framework may support stakeholders in the food supply chain for better decision-making regarding food fraud control while still preserving the privacy and confidentiality nature of these data.

## Introduction

To ensure safe and healthy food, all actors in food supply chains are collecting large amounts of food quality data (including food fraud) at the various production stages. Implementation of technologies such as drones^1^, mobile devices^2^ and Internet of Things (IoT)^3^ urge the implementation of Artificial Intelligence (AI) solutions in the food supply chain including food safety^4,5^. AI will play a key role in the digital transformation of the food supply chains in particularly in the exploitation of these vast data sources and to support the implementation of a holistic approach to ensure truly sustainable food systems^6^. It was demonstrated for food safety and food fraud that the AI method Bayesian Network (BN) is suitable to implement the holistic approach in which data from different origins and nature are integrated^4,7,8^. BNs provide transparent and interpretable probabilistic models that can handle uncertainty, incorporate prior knowledge, and make principled decisions, offering a powerful alternative to unexplainable machine learning algorithms^4,9,10^.

However, the impact of these technologies depends critically on data sharing and integration, which is one of the biggest challenges within the food supply chain^11,12^. Different data owners have different interests and priorities that hinder the incentive to share data. Data collected in the context of food safety and food fraud can be politically sensitive and considered a competitive advantage^13^, but there is also a cost associated with collecting this data. Nonetheless, there is a strong shared interest among stakeholders in food safety compliance and to prevent food fraud. It is also desirable that clear guidelines for data sharing be agreed upon. To this end, extensive negotiation among data owners is usually required to resolve issues of ownership, confidentiality, and management of the data^11^. Agreement is particularly difficult when many supply chain actors with conflicting interests are involved (e.g., competitors, control authorities, and manufacturers, etc.). An additional challenge in data sharing is that data must be described with metadata using ontologies so that it can be found by specific search engines. However, aside from FOODON^14^, few food safety and food fraud ontologies are publicly available to date, making the adoption of data sharing and integration technologies difficult. Evidence of solving these issues can be found in literature using federated learning (FL). In a federated environment, data never leave the physical location of the data owners. Instead, the algorithm (i.e., model) moves between these locations (i.e., data stations) and collects parameters from the data at the data station’s physical location. One of the main advantages of this approach is that the federated infrastructure can perform some of the “negotiation” (otherwise done by humans) automatically once data sharing policies are agreed upon. FL has recently gained attention in several domains such as life sciences^15–19^ but has, to our knowledge, not yet been explored in food domain.

In this study, the FL concept was developed to predict food fraud type through federated food fraud data stations and a BN model. It was shown that a BN model could be trained on these data stations without the data leaving the data stations and that the model performance is like a BN model developed on the same data pooled to one location. The developed FL infrastructure addresses some of the limitations that classical centralized solutions still faces such as data ownership, confidentiality, privacy, security, and increased data traffic by: (i) keeping the data locally with the data owner; (ii) requiring no exchange of raw data (iii) providing high-level data security; (iv) reducing data traffic between actors in the food chain; and (v) allowing parameter learning from all stations^20–22^.

The developed FL infrastructure has the potential to improve prediction models, benefiting all stakeholders in the food supply chain. By ensuring that data remains within the ownership of its respective database, FL effectively addresses concerns related to General Data Protection Regulation (GDPR) compliance and business sensitivity. Such a concept may stimulate the collaboration along the food supply chain, which leads to an increased trust among actors and drives efficiency across the entire food supply chain.

## Results and discussion

### Food fraud data per data station

**Fig. 1:**
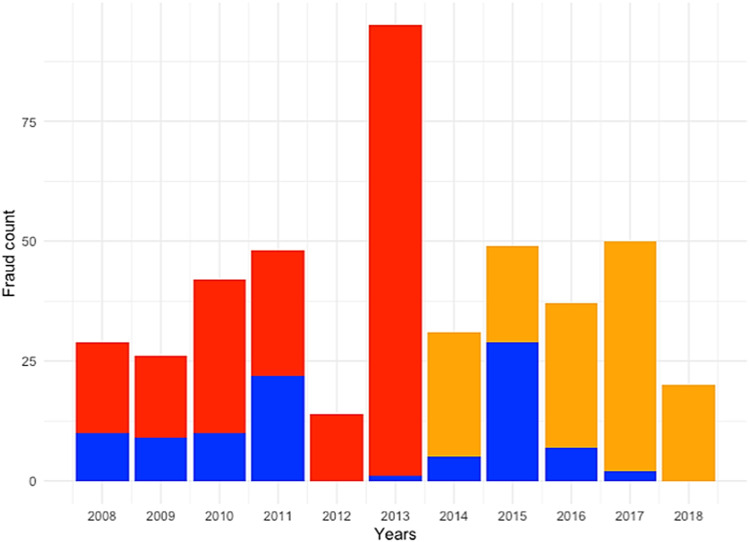
Distribution of food fraud across different data stations. Summary of data stations depicting imbalance in different fraud types on count.

Often actors in the supply chain have limited, imbalanced data available on which decisions must be made. Sharing these datasets between these actors would improve their individual models and decision making. To mimic such situation and to demonstrate how FL may solve this data sharing issue, the total available data set was separated into three incomplete data sets varying in the number of food fraud cases, years, and type of fraud (see Table 1 and Fig. 1). For example, STATION-1 contained only two types of food fraud, which are Smuggling-Mislabeling-Origin Masking (i.e., 105 observations), and Substitution-Dilution (i.e., 97 observations). All the data of STATION-1 is obtained from Rapid Alert System for Food and Feed (RASFF) from 2008 to 2013 (see Table 1). The other stations contained other types of food fraud (i.e., Artificial enhancement/Improvement) and more recent food fraud data (e.g., 2014–2018).

**Table 1:** Food fraud data available at different data stations.

Data station	Fraud type	Years	No. of cases	% Per data station
STATION-1	Smuggling-Mislabelling-Origin Masking	2008–2013	105	52%
	Substitution-Dilution		97	48%
STATION-2	Artificial enhancement/Improvement	2014–2018	1	1%
	Smuggling-Mislabelling-Origin Masking		135	94%
	Substitution-Dilution		8	6%
STATION-3	Artificial enhancement/Improvement	2008–2018	21	22%
	Smuggling-Mislabelling-Origin Masking		23	24%
	Substitution-Dilution		51	54%

### Federated BN

Within the FL infrastructure, a BN model was trained and validated to show that knowledge can be shared without source data leaving the data stations and that such sharing will benefit the decision makers in general. Two different experiments were conducted to showcase the difference in each setting to highlight the fact that despite enforcing privacy and security measures there was not significant loss in information gain compared to the situation when all data is compiled in one database and used for the BN modeling.

#### Experiment 1

**Fig. 2:**
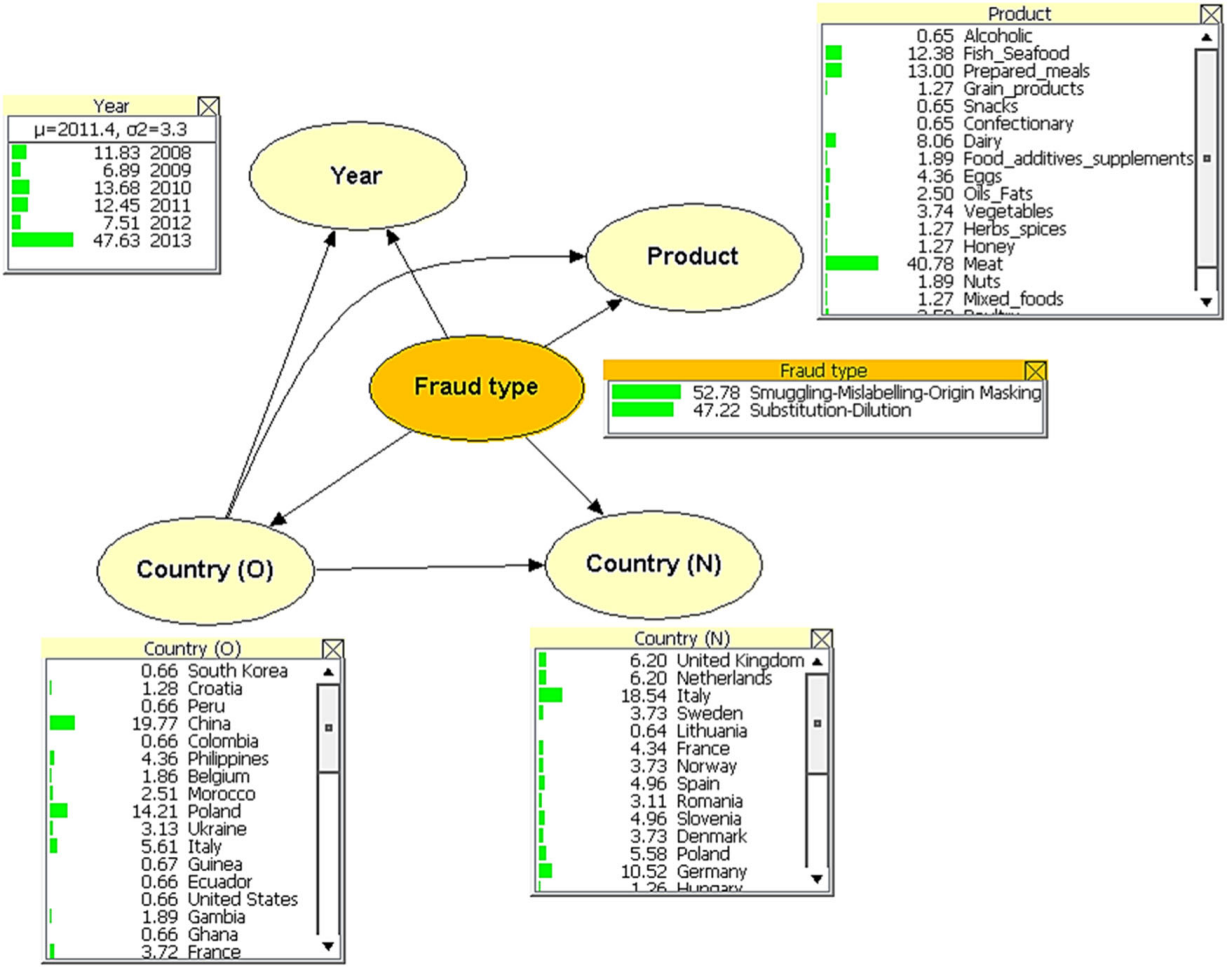
Example of the structure of the BN model for STATION-1. The nodes (ellipses in the figure) represent the indicators. The arrows indicate linkages between these nodes. The states are depicted as squares below the nodes.

A BN model was created, trained, and tested on each individual data station (i.e., individual BN model) (Table 2 and Fig. 2).

**Table 2:** AUC, average sensitivity, average specificity per station.

	STATION-1		STATION-2		STATION-3	
	Individual	Combined	Individual	Combined	Individual	Combined
AUC	0.96	0.89	0.96	0.99	0.72	0.74
Average sensitivity	0.75	0.75	0.49	0.78	0.62	0.66
Average specificity	0.69	0.63	0.83	0.69	0.58	0.59

**Fig. 3:**
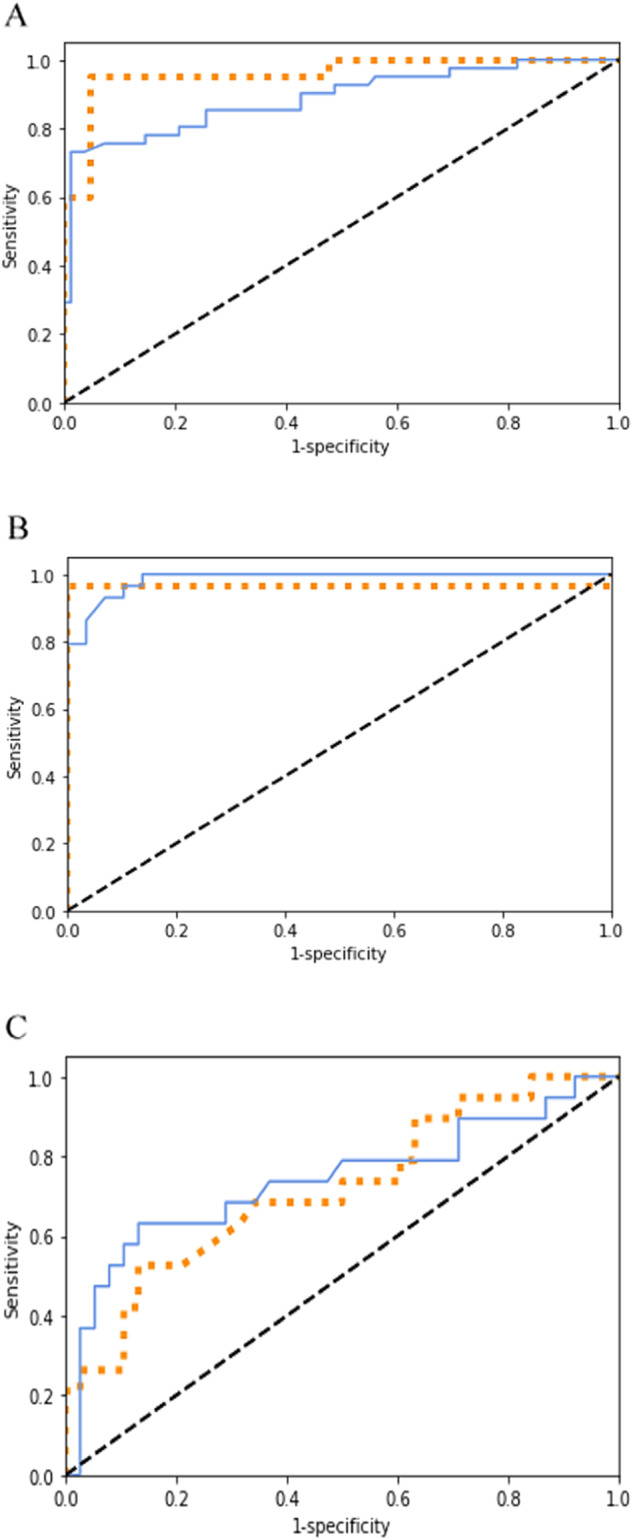
Performance of the BN models in each data station and in combined station data. **A** Performance of the BN models in data station 1 (Orange) and in combined station data (Blue). **B** Performance of the BN models in data station 2 (Orange) and in combined station data (Blue). **C** Performance of the BN models in data station 3 (Orange) and in combined station data (Blue).

ROC curves used to evaluate and compare the performance of the BN models based on the individual station data versus the combined station data for STATION-1, -2, and -3, respectively, are shown in Table 2 and Fig. 3. The overall model performance is expressed by the area under the ROC curve (AUC). The results show that the accuracy of each BN model was high for STATION-1 and -2 (i.e., AUC = 0.96), but for STATION-3 the accuracy was significantly lower (i.e., AUC = 0.72; Table 2). For the combined BN model when tested with the test data sets from the individual data stations, the accuracy was equal to 0.89 for STATION-1, 0.99 for STATION-2 and 0.74 for STATION-3 (Table 2). This shows that a larger number of data available to train/develop a BN model does not necessarily lead to higher accuracies, which can be explained in our case with the data heterogeneity (i.e., number of cases per year, different types of food fraud) in relation to food fraud type (Fig. 1). The relatively small decrease in accuracy of the combined BN compared to the individual BN in STATION-1 (AUC = 0.96 vs. AUC = 0.89) is noteworthy because the combined BN model includes three categories of food fraud, whereas the STATION-1 training data set includes only two (see Table 2). However, one should realize that the combined BN model contains more knowledge because it was trained on a broader dataset of food fraud cases than the individual datasets (different products and/or countries of origin) and therefore covers the real situation better and allows the user to make better decisions. In the case presented in this study, the owner of STATION-1 lacked the food category “artificial refinement/improvement” in the dataset, but with the “combined” BN model, the user of STATION-1 effectively gains knowledge about this type of fraud. Moreover, the ROC curves show the trade-off between sensitivity and specificity. An improvement of the combined BN model compared to the individual BN model was observed for the sensitivity parameter of performance, especially for STATION-2 (i.e., increase from 0.49 to 0.78, see Table 2). Higher sensitivity means that the combined BN model is better able to identify the food fraud cases. Nevertheless, lower specificity was also found for the combined BN model in STATION-2 (decrease from 0.83 to 0.69, see Table 2), which means that the combined BN model leads to more misclassifications of positive food fraud cases where no food fraud is present compared to the single BN model.

#### Experiment 2

**Fig. 4:**
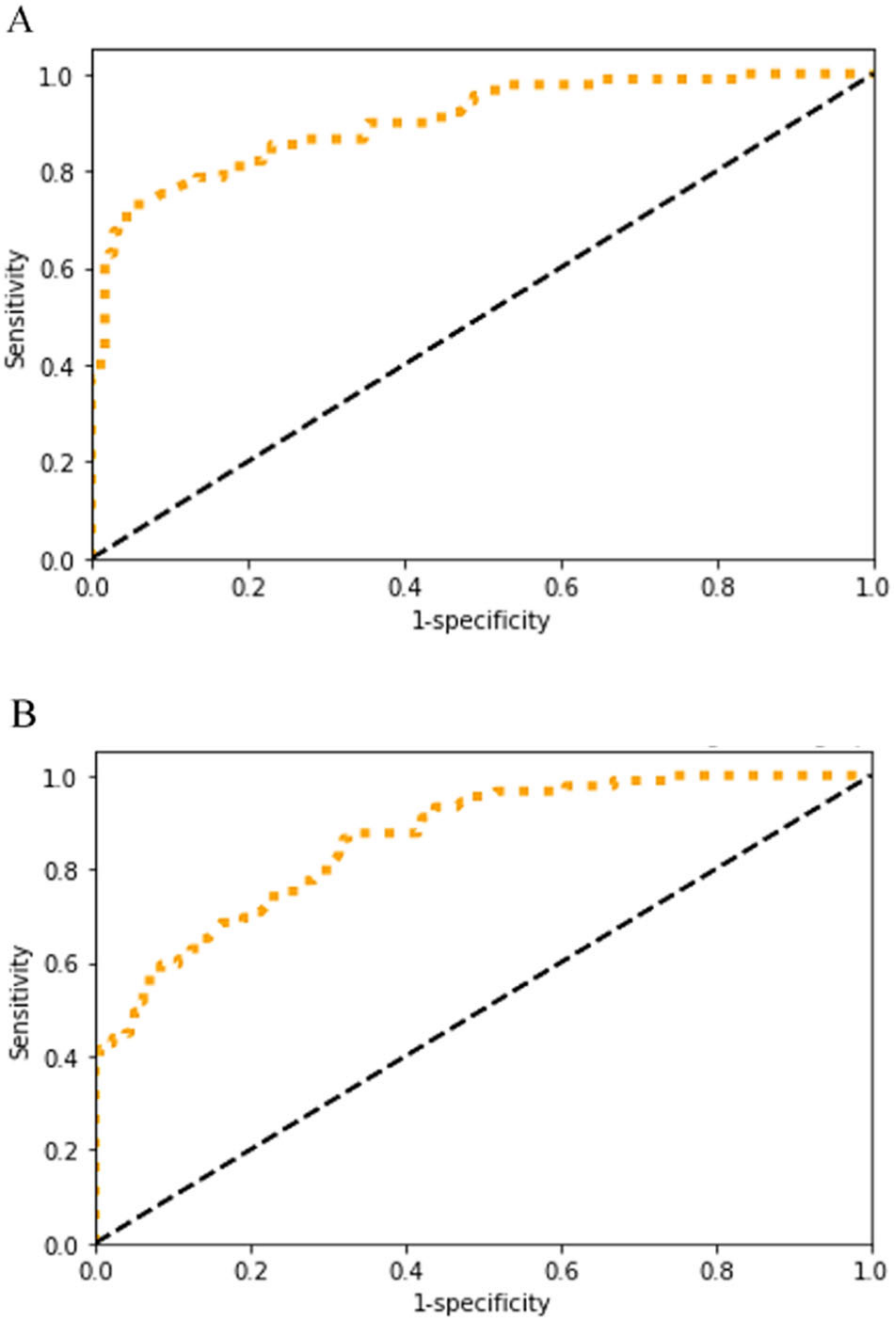
Performance of the BN models with ROC. **A** BN Model learned from the combined data in a federated manner. Micro-average ROC curve (area = 0.9) (Orange). **B** BN model learned from the combined data in a federated manner. Micro-average ROC curve (area = 0.86) (Orange).

In this setting, a BN was developed on the total dataset without using a FL infrastructure, hence the data is shared in a traditional manner (i.e., random split of the dataset, 80% for training and 20% for testing). As shown in Table 3 and Fig. 4, an AUC of this BN is 0.86 with an average sensitivity of 0.72 and an average specificity of 0.67.

**Table 3:** AUC, average sensitivity, average specificity.

	BN (80%, 20%)	BN (FL datasets combined)
AUC	0.86	0.90
Average sensitivity	0.72	0.77
Average specificity	0.67	0.64

As can be seen in Table 4, the combined AUC values with and without a FL infrastructure are very close, with an average AUC value of 0.9 for all three data stations in a federated environment compared to 0.86 without a FL infrastructure. The results show that a FL infrastructure is robust to both model performance and imbalances in the data.

**Table 4:** Metadata for data stations.

Variable name	Node name	States
Type of fraud	Fraud type	Artificial enhancement/Improvement, Smuggling-Mislabeling-Origin Masking, Substitution-Dilution
Category of product	Product	Alcoholic, Fish_Seafood, etc.
Year	Year	2008, 2009, …, 2018
Origin country	Country (O)	South Korea, Croatia, etc.
Report country	Country (N)	United Kingdom, Netherlands, etc.

An important goal of government agencies, enterprises, and research institutes using a federated infrastructure is to ensure that the security and privacy of their data comply with the GDPR and meet data protection compliance measures. In a FL infrastructure, a potential threat is running Docker images on the data stations and granting that permission without the data station owners having visibility into the source code repository of the images. Therefore, it is highly desirable that data station owners allow these images to run on their compute nodes only after validating the source code in the Docker image itself. After validation, the data owner can release this version for execution on their data. The infrastructure handles the validation of the algorithm version using a hash and Docker registry, see the details in ref. ^23^. Vantage6 uses token-based authentication and authorization for each data station to join the collaboration, while an API can be used to create such collaboration and organizations within that facility.

There are currently three main issues that are observed in a FL setup which are: data democratization, limitation on AI models, and efficiency of the tools. In most FL systems, there is not much emphasis on democratization of data, which seems to be of paramount importance for data harmonization. Current constraints in FL require that data is in a static structure in a table format (e.g., CSV) and that the order of variables in that data is maintained^24,25^. Aside from this, it is important to consider the data type, as it poses a problem when Machine Learning (ML) models use this data (e.g., a ML model might expect a number, but the value is in characters). To address some of these issues, modern data formats have been proposed, such as linked data formats like RDF, where data can be represented as triples. The advantage of this approach is that the data for ML models does not need to be in a predefined tabular structure but can be expanded as it is. However, to be able to use these data, an additional SPARQL^26^ layer must be integrated into the machine learning models, which first identifies the required variables and determines whether they are available at all from the data owners. This does not preclude still considering preprocessing steps such as missing values and other data formatting issues. When different data owners collaborate in a federated setting using same data in different contexts additional layers can be added on top of data stations where automated deciphering of data based on ontologies can be carried out using modern data standards like FHIR^27^ or OMOP^28^.

So far, there are only a limited number of AI models implemented in the FL environment. Some of them are commercial in nature such as HPE^29^, other open-source models that have just been made available in a federated environment are glm^30^. For large datasets, simple models (e.g., summary statistics) are often sufficient because big data often introduce unique statistical challenges, including scalability and storage bottleneck, noise accumulation, spurious correlation, incidental endogeneity, and measurement errors^31^. Apart from that, most complex statistical models are designed to run on single devices in a centralized setting. Modern algorithms like deep learning models need to be redesigned to leverage the power of a FL setup^32^.

Most ML models are created using languages such as Python or R. These languages allow a researcher to quickly create models for research purposes. However, these models are difficult to operationalize because they have issues with data structures, as most of them work with tabular data and are incompatible with web data formats such as JSON. While there are some packages in these languages that take care of some of the problems, they are not inherently efficient. Most of the models created in a FL environment are dockerized. Docker^33^ provides an environment that allows a ML model to be reusable and reproducible by considering all the dependencies that a ML model requires. However, since both Python and R are interpreted languages, docker images created with these languages are very large, resource intensive, and require good network bandwidth and CPU resources. Recently, efforts are being made to create models in modern compiled languages such as Golang^34^ and Rust^35^. Since the ML models created using these tools are in binary format, all dependencies are included in them, making these models more efficient both CPU and in terms of network bandwidth. In future, it is important that more ML models are built using compiled languages.

In this study, a proof of concept of FL approach was demonstrated to detect food fraud. A federated BN model was implemented in this setting that could be trained on the combined data of databases from geographically different locations, without the source data ever leaving the data stations. The principle was demonstrated for three data stations, although many more data stations can easily be linked to this infrastructure.

The results showed that the federated BN model achieved high accuracy and improved sensitivity, indicating better identification of food fraud cases. Despite the heterogeneity and imbalances of the data, the federated BN model provided a broader knowledge base, and maintained model performance while preserving privacy and security.

The FL may help to develop powerful prediction models for the benefit to all actors in the food supply chain while the data will not be leaving the database of the data owner, hence solving GDPR and business sensitivity issues. Such a concept may stimulate the collaboration along the food supply chain and lead to an increased trust among actors. In addition, making use of data from many stakeholders may also stimulate a more efficient use of resources and reduce the costs of data collection (i.e., food safety monitoring).

Overall, the findings of this study contribute to the understanding of FL infrastructure and its potential for secure and privacy-preserving knowledge sharing. This research paves the way for wider adoption of FL in various domains where data privacy is a concern and for the development of data trusts in food supply chains.

## Methods

### Federated architecture

**Fig. 5:**
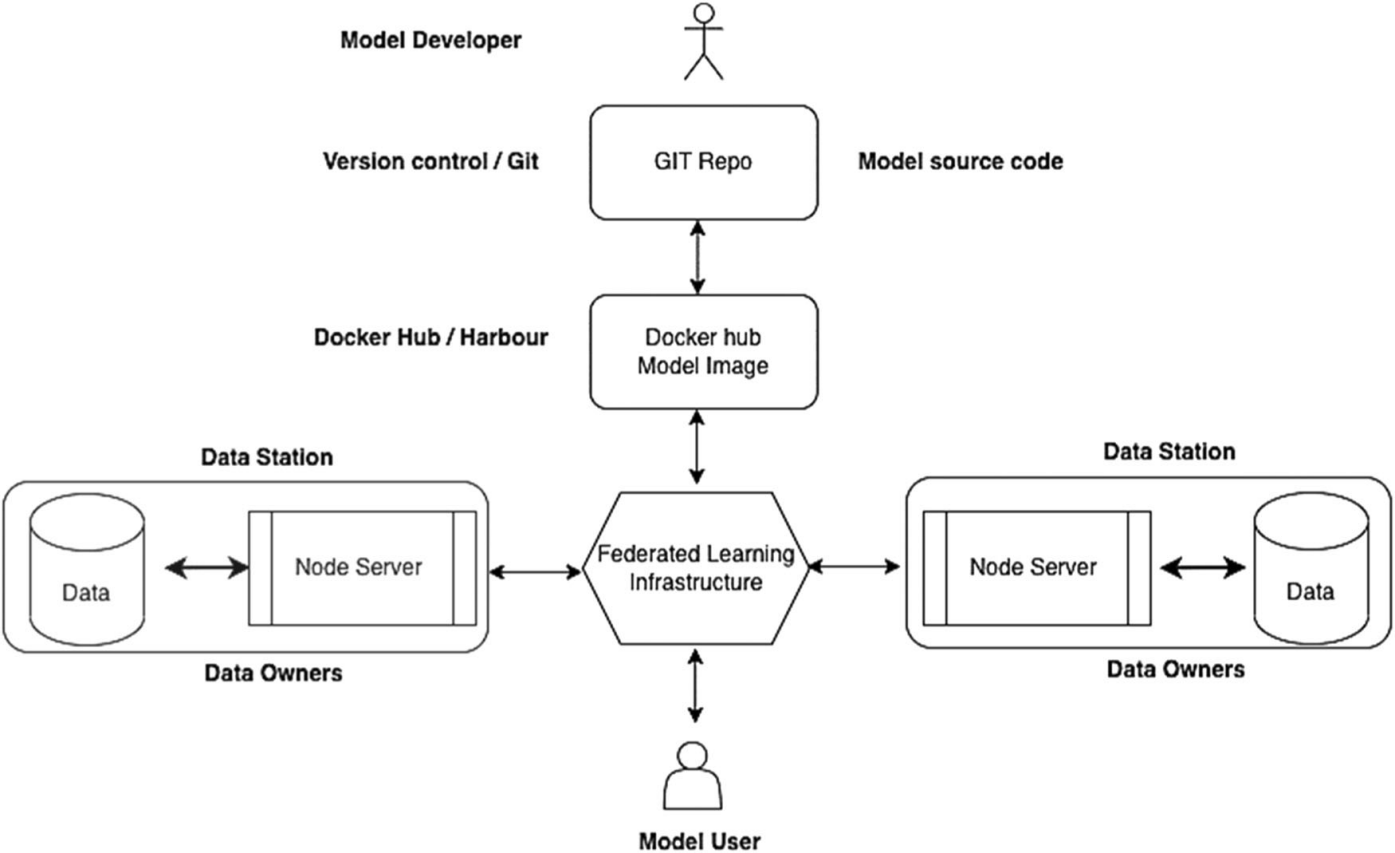
Main components of a federated architecture. Federated architecture consisting of model developers and users, FL infrastructure, and two data stations (i.e., data base of the data owner and compute node depicted as node server).

In this study, the Vantage6^36^ platform (version 2.3.4) was used, which is a FL infrastructure for secure information sharing. This central infrastructure component (authentication and message broker) was hosted at the Wageningen University & Research premise. Vantage6 enforces privacy concerns by allowing only certain algorithms to run. This ensures that data is secure even if the security of the server is compromised. Collaboration policies for data sharing were defined on the central server. To set up a federated collaboration (shown in Fig. 5), the following activities should be carried out: (i) A collaboration network is created when all participating organizations agree to work together on a particular issue. These organizations can represent any actor in the food supply chain (e.g., farmers, food industry, government agencies), who can be both owners and/or users of data and models/algorithms. This infrastructure is created from a central location where a collaboration server is established that has an integrated database that stores collaboration information and policies that determine which collaborations have access to which data stations according to those policies. An administrator makes individual organizations part of this collaboration at the central server and distributes the authentication information to all involved organizations and users; (ii) A data analysis algorithm or model learning application is created by a model developer using an appropriate language (e.g., R/Python). These scripts can be used on a particular data station that is part of this collaboration network. These scripts are typically published as Docker images in an internal Docker registry or a publicly accessible Docker registry, approved within the collaboration. All input parameters are passed to this Docker image; (iii) Any data station requesting this Docker image as part of a collaboration can have it run at the data station owner’s site if execution of that image is allowed; (iv) The computing nodes of the data stations return the results after the algorithm has been executed. These results are sent to the central server, from which the model users can retrieve the results.

Each data station is connected to various components that enable the entire workflow of data storage and computation in a secure environment. These components include the data volume (i.e., local storage for data in csv files or a database), the Docker daemon (i.e., software to run Docker images), the algorithm container (i.e., compute node running the Docker image of the algorithm), CLI (i.e., command line interface to start, stop and debug nodes using log files or configuring new nodes), and a set of configuration files consisting of data policies and API keys that connect this data station to the federated server it belongs.

## Experimental case study: food fraud

### Food fraud data and data stations

Data for this study is derived from two major sources: the European Union (EU) Rapid Alert for Food and Feed (RASFF)^37^ database and the United States (US) Economic Motivation Adulteration (EMA) database from the period these were publicly available^7^.

**Fig. 6:**
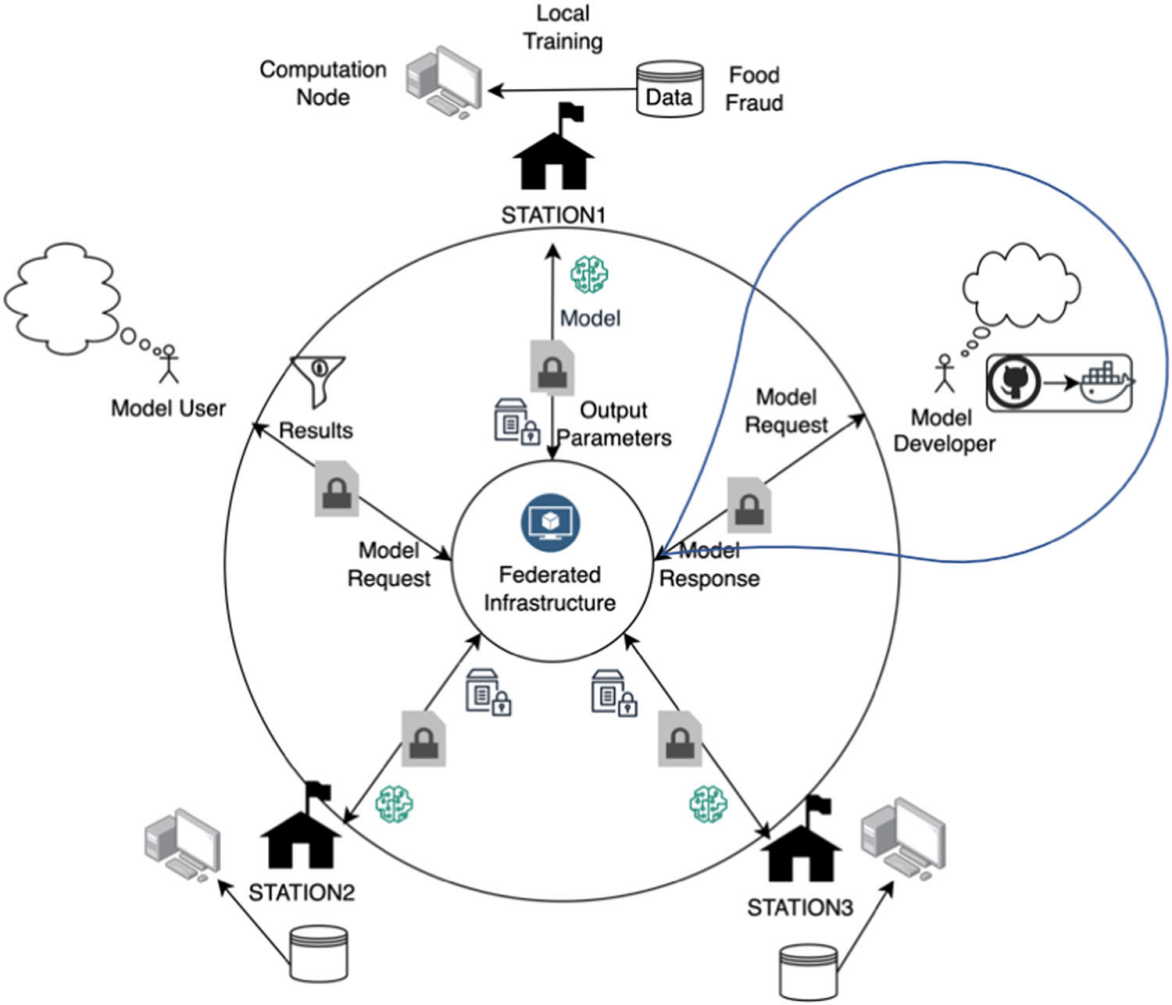
Conceptual framework of collaboration aimed at prediction of food fraud. The model users first train their model on their own local data stations using a BN model (embedded inside a docker image). The trained model parameters are then transferred to the central server. These parameters are subsequently retrieved back by model users in a secured setting via the central server for each station to generate a combined BN model which contains information from each data station.

The collected data was separated in three portions and placed in different databases, representing a hypothetical situation of three data owners. For each dataset, a data station was prepared and hosted at a different geographic location in the Netherlands, namely Wageningen (STATION-1), Maastricht (STATION-2), and Utrecht (STATION-3) (Fig. 6). STATION-1 contained RASFF data from 2008 to 2013 (i.e., 202 observations), STATION-2 contained RASFF data from 2014 to 2018 (i.e., 144 observations), while STATION-3 contained EMA data from 2008 to 2017 (i.e., 95 observations). For each of these datasets, food fraud type, product category, year, origin country and the control country were selected to be used for data training (i.e., the BN model). The meanings, corresponding nodes, and states of the variables of the BN model are listed in Table 4.

All metadata belonging to each of these stations is made available on an internally hosted FAIR Data Point^38^ that can be accessed at WUR^39^.

To demonstrate the applicability of the infrastructure, different data formats were used. The data located at STATION-1 and STATION-2, containing RASFF food fraud notifications, were in CSV format and the data located at STATION-3, containing EMA food fraud notifications, was in RDF format.

**Fig. 7:**
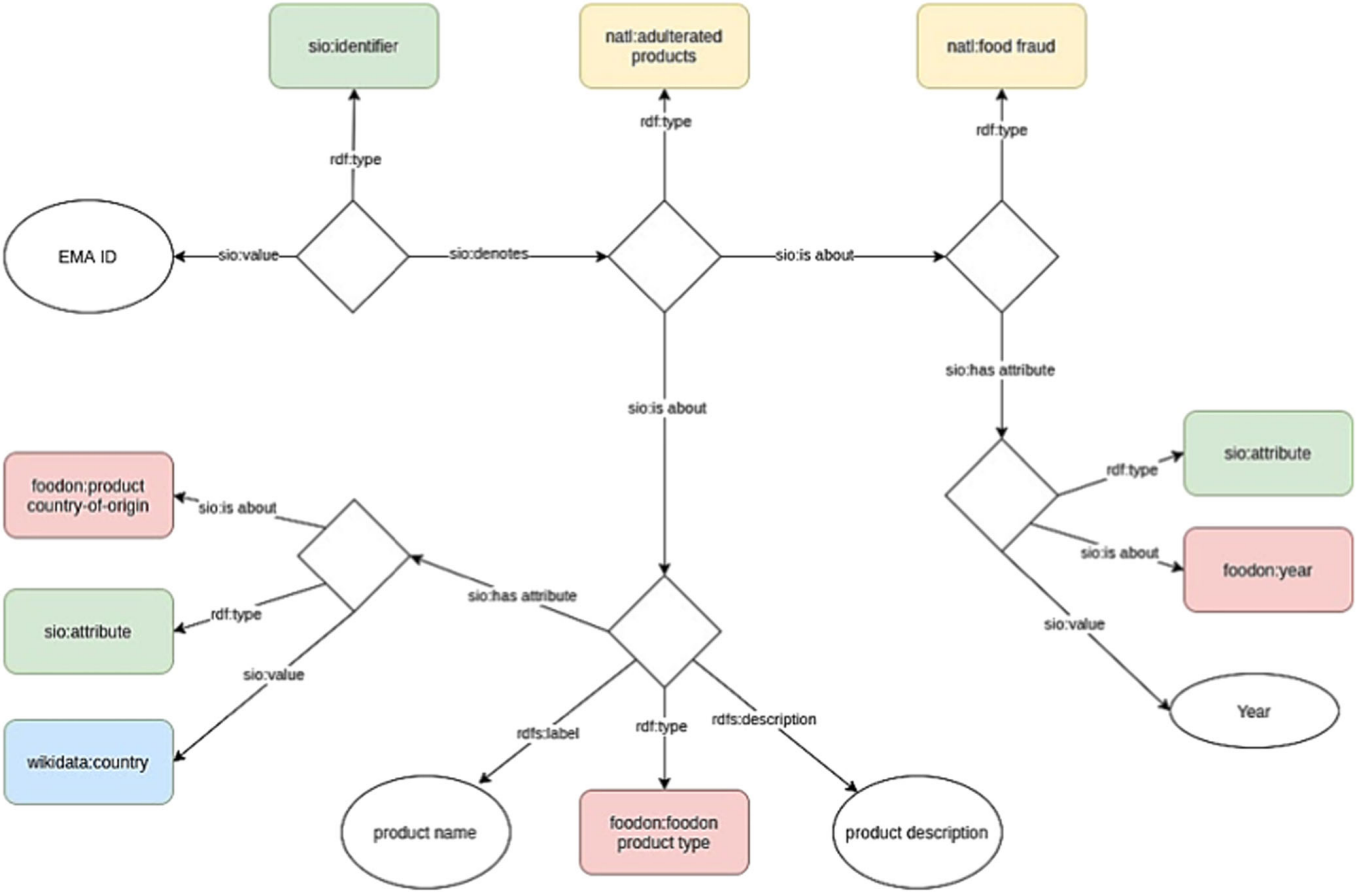
RDF model of EMA data as hosted on STATION-3 node. FoodOn ontology (red); semanticscience integrated ontology (green); national agriculture library thesaurus (yellow); wikidata entries (blue).

The data from STATION-3 were in a semantically interoperable RDF format and are shown in Fig. 7, including their metadata. This data model was created using ontologies such as the National Agricultural Library Thesaurus, FOODON and Semantic Science Integrated Ontology (SIO), and Wikidata. To date, there is no specific ontology for food fraud and only minimal information standards exist that have not yet been formalized. Therefore, we have relied on generic terminologies that would increase the use(ability) of them for the community. The AgroPortal^40^ was used to search for these ontologies, which contains various ontologies in the field of agricultural and plant sciences. This RDF data model was then converted to a CSV format by STATION-3 to be hosted on STATION-3 nodes, because the BN model only can consume data from CSV.

### Federated BN model

In this work, we implemented a BN model, in a federated environment. The R package “bnlearn”^41^ was used to apply “Bayesian network analysis” to the data. The algorithm is encapsulated in a Docker image and the R algorithm library of Vantage6 is used. This library contains auxiliary functions for input/output between the infrastructure and the algorithm. This allows developers to focus on implementing the algorithm and worry less about the infrastructure-specific code. The source code for this library can be found in the repository (see “Data availability” section).

A BN model was developed that automatically divides the dataset into a training (80%) and a test dataset (20%) and learns using a data framework. The algorithms use a standard CSV file as input. Tree-Augmented Naive Bayes (TAN) was applied to learn the structure of BN on each of the training datasets for the variable “Fraud” (i.e., the fraud type). Once the structure was known, the parameters were estimated using the bn.fit function with the “Bayes” method to derive the three BN models. The model was trained using characteristics such as the product susceptible to fraud (e.g., eggs, oils, oily fish, and seafood), the year (e.g., when it was first reported), the origin (e.g., which country the food came from), and the type of fraud (e.g., substitution, mislabeling, etc.) to make predictions about “fraud”. All data stations that provided this data had these variables in common.

Finally, the predicted “fraud” type was compared to the observed “fraud” type recorded in the test data sets to obtain the prediction accuracy, sensitivity, and specificity. This model provides the results in web-enabled json format.

### Description of the experiments

To demonstrate the operation of the developed federated infrastructure, two experiments were conducted to (a) first, test how a BN model trained on the aggregate dataset of all three data stations in a federated environment performs on the test dataset of each of the data stations separately. Second, (b) we tested how a model from BN, trained on the aggregated dataset of all three data stations without a federated environment, performs on the aggregated test dataset.

#### Experiment 1

A BN model was developed, trained, and evaluated on each individual data station, resulting in multiple individual BN models. The performance of each individual BN model was then compared to that of a combined BN model, which was trained on the aggregated training data from all data stations. Subsequently, the performance of the combined BN model was tested on the individual test data from each respective data station.

#### Experiment 2

The objective of this experiment was to examine and comprehend differences between the two approaches: with and without federated settings. In this case, a BN was constructed using the entire dataset without utilizing a FL infrastructure. Consequently, the BN was based on a random split of the dataset, allocating 80% for training and 20% for testing purposes.

### Reporting summary

Further information on research design is available in the Nature Research Reporting Summary linked to this article.

### Supplementary information

Reporting Summary

## Data Availability

Datasets used are available in the following link: https://zenodo.org/record/8220868.

## Code availability

Source code: (a) Vantage6 library: https://vantage6.ai. (b) Bayesian Network Algorithm: https://zenodo.org/record/8220868. (c) Docker image: harbor2.vantage6.ai/wur/vtg.wur.
