# Author Correction: Applying federated learning to combat food fraud in food supply chains

**DOI:** 10.1038/s41538-023-00232-z

**Published:** 2023-10-19

**Authors:** Anand Gavai, Yamine Bouzembrak, Wenjuan Mu, Frank Martin, Rajaram Kaliyaperumal, Johan van Soest, Ananya Choudhury, Jaap Heringa, Andre Dekker, Hans J. P. Marvin

**Affiliations:** 1 Industrial Engineering & Business Information Systems, University of Twente, Enschede, The Netherlands; 2 Wageningen Food Safety Research, Akkermaalsbos 2, 6708 WB Wageningen, The Netherlands; 3 Information Technology Group, Wageningen University and Research, Wageningen, The Netherlands; 4 Netherlands Comprehensive Cancer Organization (IKNL), Eindhoven, The Netherlands; 5 Department of Human Genetics, Leiden University Medical Center, Leiden, The Netherlands; 6 Brightlands Institute for Smart Society, Faculty of Science and Engineering, Maastricht University, Heerlen, The Netherlands; 7 Department of Radiation Oncology (Maastro), GROW School for Oncology and Reproduction, Maastricht University Medical Centre, Maastricht, The Netherlands; 8 Centre for Integrative Bioinformatics (IBIVU), VU University Amsterdam, Amsterdam, The Netherlands; 9 Department of Research, Hayan Group, Rhenen, The Netherlands

**Keywords:** Risk factors, Chemical safety

Correction to: *npj Science of Food* 10.1038/s41538-023-00220-3, published online 01 September 2023

“In this article the affiliation details for Anand Gavai, Yamine Bouzembrak, Hans J. P. Marvin were incorrectly given as ‘Anand Gavai^1^, Yamine Bouzembrak^2^, Hans J. P. Marvin^9^ ‘, but should have been ‘Anand Gavai^1,2^, Yamine Bouzembrak^2,3^, Hans J. P. Marvin^2,9^’. The original article has been corrected.”

